# Feasibility of three times weekly symptom screening in pediatric cancer patients

**DOI:** 10.1186/s12885-022-10400-1

**Published:** 2023-01-03

**Authors:** Maryann Calligan, Lauren Chakkalackal, Grace Dadzie, Cassandra Tardif-Theriault, Sadie Cook, Emily Vettese, Dilip Soman, Susan Kuczynski, Tal Schechter, L. Lee Dupuis, Lillian Sung

**Affiliations:** 1 Program in Child Health Evaluative Sciences, Peter Gilgan Centre for Research and Learning, The Hospital for Sick Children, 686 Bay Street, Toronto, Ontario M5G 0A4 Canada; 2 Rotman School of Management, University of Toronto, 105 St. George Street, Toronto, ON M5S 3E6 Canada; 3 Ontario Parents Advocating for Children with Cancer (OPACC), 99 Citation Drive, Toronto, ON M2K 1S9 Canada; 4 Division of Haematology/Oncology, The Hospital for Sick Children, 555 University Avenue, Toronto, ON M5G 1X8 Canada; 5 Department of Pharmacy, The Hospital for Sick Children, and Leslie Dan Faculty of Pharmacy, University of Toronto, The Hospital for Sick Children, 555 University Avenue, Toronto, ON M5G 1X8 Canada

**Keywords:** Symptom screening, Quality of life, Pediatric, Oncology

## Abstract

**Objective:**

Primary objective was to determine the feasibility of three times weekly symptom reporting by pediatric cancer patients for eight weeks.

**Methods:**

We included English-speaking patients 8–18 years of age with cancer. Patients were sent reminders by text or email to complete Symptom Screening in Pediatrics Tool (SSPedi) three times weekly for eight weeks. When patients reported at least one severely bothersome symptom, the symptom report was emailed to the primary healthcare team. Patient-reported outcomes were obtained at baseline, week 4 ± 1 and week 8 ± 1. Symptom documentation, intervention provision for symptoms and unplanned healthcare encounters were determined by chart review at weeks 4 and 8. The primary endpoint was feasibility, defined as at least 75% patients achieving adherence with at least 60% of SSPedi evaluations. We planned to enroll successive cohorts until this threshold was met.

**Results:**

Two cohorts consisting of 30 patients (cohort 1 (*n* = 20) and cohort 2 (*n* = 10)) were required to meet the feasibility threshold. In cohort 1, 11/20 (55%) met the SSPedi completion threshold. Interventions applied after cohort 1 included engaging parents to facilitate pediatric patient self-report, offering mechanisms to remember username and password and highlighting potential benefits of symptom feedback to clinicians. In cohort 2, 9/10 (90%) met the SSPedi completion threshold and thus feasibility was met. Patient-reported outcomes and chart review outcomes were obtained for all participants in cohort 2.

**Conclusions:**

Three times weekly symptom reporting by pediatric patients with cancer for eight weeks was feasible. Mechanisms to enhance three times weekly symptom reporting were identified and implemented. Future studies of longitudinal symptom screening can now be planned.

**Supplementary Information:**

The online version contains supplementary material available at 10.1186/s12885-022-10400-1.

## Background

Substantial gains in survival among pediatric patients with cancer has led to increasing attention focused on improving quality of life and controlling symptoms [1]. Pediatric oncology patients experience a high prevalence of severely bothersome symptoms while receiving cancer treatments [2]. We know from studies in adult cancer patients that routine collection of patient-reported outcomes improves patient-clinician communication [3], reduces distress [4] and improves quality of life [5, 6]. Consequently, in adult oncology practice, screening and assessment of symptoms are important priorities [7–10]. In contrast to these advances in adult cancer care, efforts in children are limited [11, 12].

**Fig. 1:**
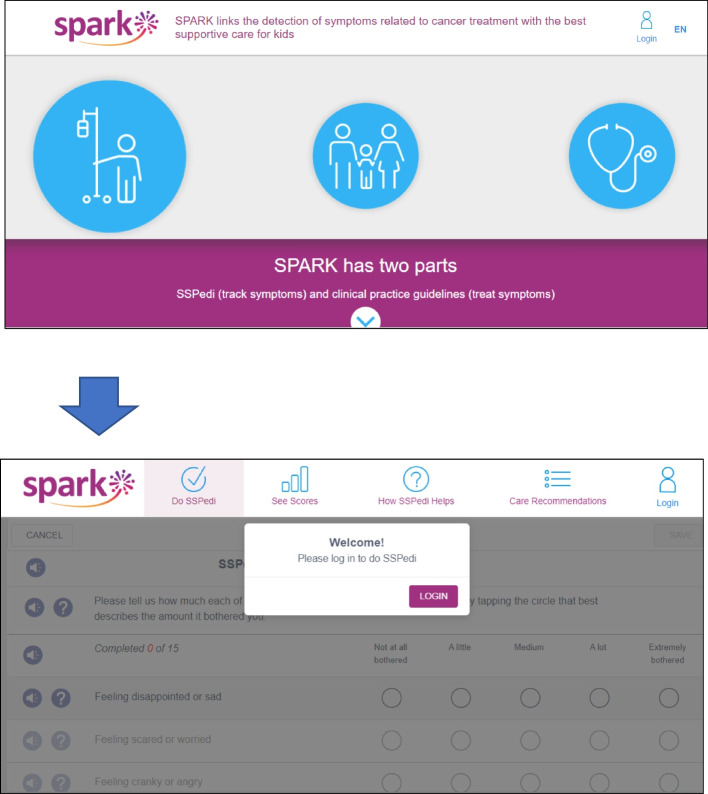
SPARK Landing Page and Patient Portal

In order to address this gap, we created the Symptom Screening in Pediatrics Tool (SSPedi), which is a self-report symptom screening and assessment tool for pediatric patients 8–18 years of age receiving cancer treatments. Building on SSPedi, we then developed Supportive care Prioritization, Assessment and Recommendations for Kids (SPARK), which is a web-based platform that consists of a symptom screening component centered on SSPedi and a supportive care clinical practice guideline component (Fig. 1) [13]. SPARK provides reminders for pediatric patients to complete symptom screening by text or email. When the patient reports at least one severely bothersome symptom, SPARK sends an email to the primary healthcare team with the patient’s symptom report.

Creation of the symptom screening tool and web application are necessary steps but are not sufficient in themselves to enable routine utilization. Identifying approaches to facilitate symptom screening in clinical practice is a required step toward improving symptom control. We previously established the feasibility of daily completion of symptom screening for five days among pediatric cancer patients who were either admitted to hospital or seen in clinic for five consecutive days [14, 15]. We next planned to address longitudinal completion of symptom screening among pediatric cancer patients over a longer period of time, including when patients were at home. Consequently, the primary objective was to determine the feasibility of three times weekly symptom reporting by pediatric patients using the SPARK platform for eight weeks. Feasibility threshold was defined as 75% of patients achieving adherence with at least 60% of SSPedi evaluations. Secondary objectives were to describe patient-reported outcomes, symptom documentation, intervention provision for symptoms and unplanned healthcare encounters.

## Methods

This was an open label, single center feasibility study enrolling pediatric cancer patients at The Hospital for Sick Children in Toronto, Canada. This study was approved by the Research Ethics Board at The Hospital for Sick Children and all participants provided informed consent or assent (as appropriate). This study was registered with clinicaltrials.gov on 19/02/2019 (NCT04275102).

### Subjects

We included children and adolescents with cancer who were 8–18 years of age at enrollment who had received or who had a plan to receive any cancer treatment and who were English-speaking. Exclusion criteria were cognitive disability or visual impairment (even with corrective lens) that precluded use of SPARK.

### Procedures

Potential participants were identified by research staff and recruited from the inpatient wards and outpatient clinics. Patients required a device to access SPARK to complete SSPedi; the device could be a smart phone, tablet or computer. If the patient did not have access to a device, tablets were available for loan. For consenting patients, demographic information was obtained from the patient or the patient’s health records. Information included sex, age at enrollment, race, diagnosis, metastatic disease, treatments received (chemotherapy, surgery, radiotherapy or hematopoietic stem cell transplantation), inpatient status at enrollment, time from diagnosis and patient’s native/spoken language(s).

Consenting participants were added to the SPARK platform by research team members. Information recorded in SPARK included whether the patient preferred to receive reminders by email, text or both, preferred days and times for the three times weekly reminders and the names and email addresses of the primary healthcare team who would receive SPARK reports. SPARK reports were sent if the patient reported at least one severely bothersome symptom (SSPedi score of 3 or 4 on the 5-point Likert scale ranging from 0 to 4). The SPARK reports included the patient’s SSPedi symptoms depicted graphically and links to pediatric cancer supportive care guidelines. Healthcare professionals receiving SPARK reports had to have an email domain that matched the institutional email domain as one approach to protecting patient privacy. Other approaches were that SPARK underwent a security and privacy evaluation, and no one outside of the enrolling institution had access to personal health information.

At enrollment, a clinical research associate taught the patient to expect to receive reminders to complete SSPedi based on their preferred mechanism (email or text) and how to log-in to SPARK to complete SSPedi upon receiving these reminders. To log-in to SPARK, the patient had to choose a username and password. Patients were given a reminder information sheet including the days and times of their reminders as well as their username and password. In contrast to the teaching provided to patients, healthcare team recipients of SPARK reports did not receive formal training in interpreting the report as our previous research showed these reports were easy to understand [13].

A clinical research associate monitored adherence with SSPedi assessments. If a participant missed two SSPedi assessments in a row, they were contacted in person or by email to ensure they were receiving their reminders and asked if they wanted to change their reminder schedule or reset their SPARK password. Active intervention lasted for eight weeks starting from the date of enrollment.

Patient-reported outcomes (SSPedi, the PROMIS fatigue scale, and the PedsQL 3.0 Acute Cancer Module) were obtained by a clinical research associate at baseline, week 4 ± 1 and week 8 ± 1. They were collected either in person during a clinic visit or hospital admission, or remotely by telephone or web conferencing platform. Symptom documentation, intervention provision for symptoms and unplanned healthcare encounters were determined by chart review at weeks 4 and 8. Interventions provision included pharmacological interventions, non-pharmacological interventions (such as physical activity) and consultation services.

### Outcomes

The primary endpoint was feasibility, defined as at least 75% patients achieving adherence with at least 60% of SSPedi evaluations (more specifically 15 of 24 SSPedi assessments).

Secondary endpoints were potential efficacy endpoints for future randomized trials. These included SSPedi scores, fatigue, quality of life, symptom documentation and intervention provision, and unplanned healthcare encounters (emergency department visits, unplanned clinic visits or unplanned hospital admissions).

The total SSPedi score is the sum of each of the 15 SSPedi item’s Likert scores, resulting in a total score that ranges from 0 (no bothersome symptoms) to 60 (worst bothersome symptoms). The recall period is yesterday or today. The total SSPedi score is reliable, valid and responsive to change in pediatric patients 8–18 years of age with cancer or hematopoietic stem cell transplant recipients [2]. We also reported the number of patients reporting severely bothersome symptoms, defined as those reporting a symptom was “a lot” or “extremely” bothersome (score of 3 or 4 on the 5-point Likert scale ranging from 0 to 4).

Fatigue was measured using the PROMIS fatigue scale. The PROMIS fatigue item bank measures the experience of fatigue and the impact of fatigue on activities. The recall period is the last 7 days. A standardized score is provided where 50 ± 10 represents the mean and standard deviation of a United States general population [16]. A higher PROMIS score represents more of the concept being measured and consequently, it reflects worse fatigue. It is reliable and valid in pediatric patients 5–18 years of age with cancer [17]. Quality of life was measured using the PedsQL 3.0 Acute Cancer Module [18]. The 7-day recall version was used. This measure is a multidimensional instrument that is reliable and valid in pediatric patients with cancer [18]. It assesses pain and hurt, nausea, procedural anxiety, treatment anxiety, worry, cognitive problems, perceived physical appearance and communication. The total score is the sum of all the items over the number of items answered. Scores are transformed on a 0 to 100 scale where higher scores indicate better health.

Symptom documentation and intervention provision were abstracted at weeks 4 and 8. The health records were examined over a three-day window between the day prior and the day following the assessment day where the assessment day was the date in which the PROMIS fatigue scale was obtained. All documentation including notes, orders such as medications and flowsheets were included in the review process. We abstracted whether each SSPedi symptom was documented within each of the two abstraction windows (weeks 4 and 8). We also abstracted whether an intervention was provided for each SSPedi symptom within each of the two abstraction windows. Clinical research associates were trained using a standard procedure to identify documentation of symptoms including synonyms and interventions as previously described [19]. Two trained clinical research associates independently abstracted symptom documentation and intervention provision. Any discrepancies were resolved by consensus and if consensus could not be achieved, a third trained clinical research associate adjudicated.

Finally, we identified the number of unplanned healthcare encounters defined as emergency department visits, unplanned clinic visits or unplanned hospital admissions between enrollment (excluding enrollment day) and day 56. Planned clinic visits and admissions were defined as those predetermined at the time of treatment plan initiation. All other healthcare encounters were considered unplanned. We reviewed the health records to determine whether any of the 15 SSPedi symptoms were documented during emergency department visits, unplanned clinic visits or at presentation for an unplanned admission.

### Sample size and statistics

We planned to initially enroll 20 participants and if feasibility metrics were not met at that time, to enroll successive cohorts of 10 participants until feasibility metrics were met or a maximum of 60 participants had been enrolled. After each cohort, the study team met to discuss the results and decide whether modifications to the approach were required and whether feasibility metrics were met. All statistics were descriptive.

## Results

**Fig. 2:**
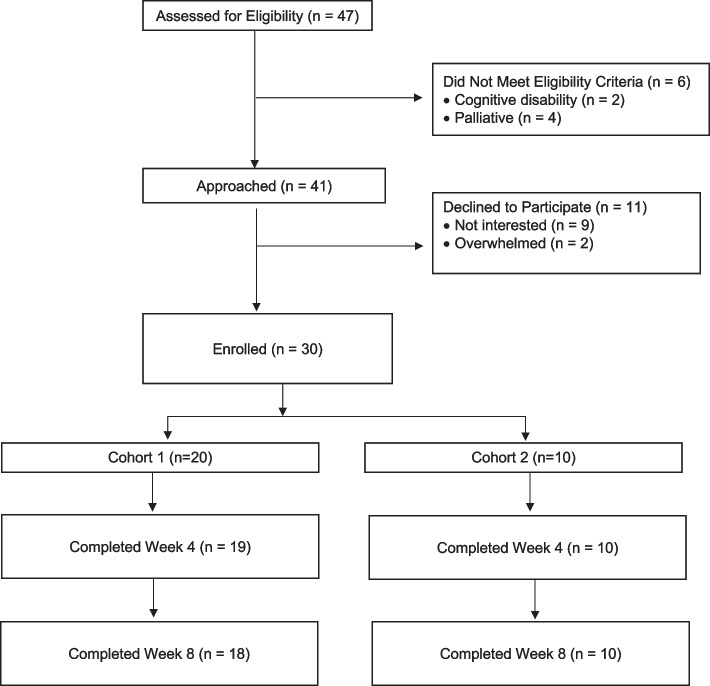
Flowchart of Participant Identification and Selection

Fig. 2 shows the flow diagram of patient identification, and reasons for exclusion and declining participation. Two cohorts consisting of the initial 20 patients (cohort 1) and one subsequent cohort of 10 patients (cohort 2) were required to meet the feasibility metrics. Consequently, we enrolled 30 patients in total between March 5 and November 25, 2021. Of the 30 patients, one came off study prior to the week 4 assessment and withdrew permission for chart review for the weeks 4 and 8 endpoints. Week 4 endpoints were obtained for all remaining 29 patients and week 8 patient-reported outcomes were obtained for 28 patients (one missed assessment). Table 1 shows patient characteristics by patient cohort. Overall, 10 (33.3%) were 8–12 years of age and 20 (66.7%) were 13–18 years of age. The most common diagnosis type was leukemia.

**Table 1:** Participant Demographic Characteristics

Characteristic	Total No. (%)	Cohort 1	Cohort 2
**Patient Characteristics**	*n* = 30	*n* = 20	*n* = 10
Male	19 (63.3%)	14 (70.0%)	5 (50.0%)
Age in Years			
8–12	10 (33.3%)	8 (40.0%)	2 (20.0%)
13–18	20 (66.7%)	12 (60.0%)	8 (80.0%)
White	17 (56.7%)	9 (45.0%)	8 (80.0%)
Diagnosis			
Leukemia	11 (36.7%)	9 (45.0%)	2 (20.0%)
Lymphoma	7 (23.3%)	3 (15.0%)	4 (40.0%)
Solid tumor	7 (23.3%)	4 (20.0%)	3 (30.0%)
Brain tumor	5 (16.7%)	4 (20.0%)	1 (10.0%)
Metastatic Disease	9 (30.0%)	5 (25.0%)	4 (40.0%)
Relapse	2 (6.7%)	2 (10%)	0 (0%)
Treatments Received			
Chemotherapy	29 (96.7%)	19 (95.0%)	10 (100.0%)
Surgery	1 (3.3%)	0 (0.0%)	1 (10.0%)
Radiotherapy	1 (3.3%)	0 (0.0%)	1 (10.0%)
Stem cell transplantation	2 (6.7%)	2 (10%)	0 (0%)
Inpatient at Enrollment	8 (26.7%)	5 (25.0%)	3 (30.0%)
Date of Diagnosis			
< 6 months before enrollment	20 (66.7%)	10 (50%)	10 (100%)
6 to 12 months before enrollment	5 (16.7%)	5 (25%)	0 (0%)
> 12 months before enrollment	5 (16.7%)	5 (25.0%)	0 (0%)
English as First Language	24 (80.0%)	15 (75.0%)	9 (90.0%)
Other Languages Spoken			
French	3 (10%)	2 (10%)	1 (10%)
Tamil	3 (10%)	2 (10%)	1 (10%)
Other*	8 (26.7%)	7 (35%)	1 (10%)

*Other languages included: Urdu (*n* = 2), Arabic (*n* = 2), Spanish (*n* = 1), Mandarin (*n* = 1), Russian (*n* = 1), Portuguese (*n* = 1), Brushaski (*n* = 1)

Additional file 1: Appendix 1 illustrates more specific information about the feasibility metrics. While the median number of SSPedi completed was similar in cohort 1 (21 SSPedi completed) and cohort 2 (22 SSPedi completed), the number that met the 60% threshold was only 11/20 (55%) in cohort 1. Thus, the 75% pre-determined threshold was not met.

Table 2 summarizes the challenges identified and the interventions instituted to address them. The challenges were: (1) patients unwilling to complete SSPedi on their own; (2) forgetting SPARK username and password; (3) unaware of potential benefits of symptom feedback to primary healthcare team; and (4) unclear on how to use SPARK on their own device. Interventions to address these challenges included the following: (1) engaging parents to enable pediatric patient self-reporting of symptoms; (2) suggesting strategies to help them remember their username and password; (3) highlighting that the primary healthcare team will receive a SPARK report if the patient reports at least one severely bothersome symptom; and (4) training patients and parents to use SPARK on their own device. After instituting these approaches, 9/10 patients in cohort 2 met the 60% threshold and thus, feasibility was established.

**Table 2:** Challenges and Interventions to Improve Adherence with Symptom Screening

Challenge	Intervention
Patient less invested in study than parent Patient unwilling to complete SSPedi on their own	Engage parent to enable pediatric patient self-reporting of symptoms by: • Present the option to have parent receive reminders in addition to the patient • Parent can sign-in to SPARK on behalf of the patient and hand the device to the patient to self-report symptoms using SSPedi
Patient and parent forgetting SPARK username and password	Encourage patient and parent use the following to remember the SPARK username and password: • Take a picture of the reminder information sheet (includes their username and password) using their device (such as smart phone) • Write down their username and password in their device • Save their username and password on their device
Patient and parent unaware of potential benefits of study	Highlight to patient and parent that the primary healthcare team will receive a SSPedi report by email if the patient reports at least one symptom that is “a lot” or “extremely” bothersome • Inform patient and parent at point of study introduction • Remind patient and parent at week 4 time point
Patient and parent unclear on how to use the SPARK platform on their own device	Train patient and parent together to use SPARK on their own device • Have patient or parent navigate to the SPARK website on their own device • Suggest patient or parent bookmark SPARK website on their own device

**Fig. 3:**
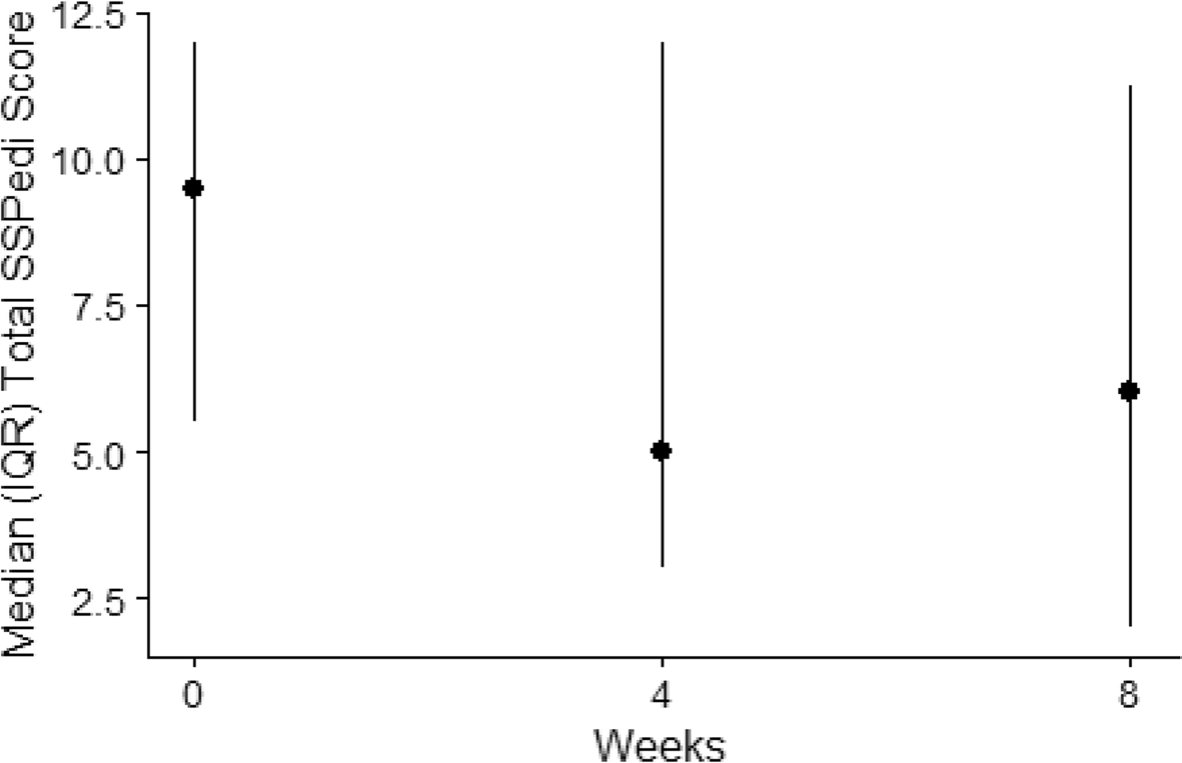
Total SSPedi Score by Assessment Time Point

Table 3 describes SSPedi total scores and the number reporting severely bothersome symptoms at baseline, week 4 and week 8. Median SSPedi scores (interquartile range (IQR)) at baseline, week 4 and week 8 were 10 (6–12), 5 (3–12) and 6 (2–11) (Fig. 3). The number of patients reporting at least one severely bothersome symptom at baseline, week 4 and week 8 were 14 (46.7%), 7 (24.1%) and 4 (14.3%). Table 3 also illustrates median PROMIS fatigue scale scores and PedsQL 3.0 Acute Cancer Module scores by time point. The most common severely bothersome symptoms reported at baseline were “feeling tired” (5, 16.7%) and “feeling more or less hungry than you usually do” (5, 16.7%).

**Table 3:** SSPedi and Patient-reported Outcomes by Assessment Time Point (*N* = 30)

	Baseline	Week 4	Week 8
	*n* = 30	*n* = 29	*n* = 28
**SSPedi Outcome Scores**			
Median SSPedi score (IQR)	10 (6 to 12)	5 (3 to 12)	6 (2 to 11)
Median Minutes to Complete (IQR)	1.6 (1.4 to 2.0)	0.9 (0.8 to 1.7)	1.0 (0.5 to 1.2)
Number Severely Bothersome (%)*			
Any symptom	14 (46.7%)	7 (24.1%)	4 (14.3%)
Feeling disappointed or sad	1 (3.3%)	0 (0.0%)	1 (3.6%)
Feeling scared or worried	1 (3.3%)	1 (3.4%)	0 (0.0%)
Feeling cranky or angry	0 (0.0%)	1 (3.4%)	0 (0.0%)
Problems with thinking or remembering things	1 (3.3%)	0 (0.0%)	0 (0.0%)
Changes in how your body or face look	2 (6.7%)	0 (0.0%)	1 (3.6%)
Feeling tired	5 (16.7%)	4 (13.8%)	0 (0.0%)
Mouth sores	3 (10%)	1 (3.4%)	0 (0.0%)
Headache	1 (3.3%)	2 (6.9%)	0 (0.0%)
Hurt or pain (other than headache)	1 (3.3%)	0 (0.0%)	0 (0.0%)
Tingly or numb hands or feet	1 (3.3%)	1 (3.4%)	1 (3.6%)
Throwing up or feeling like you may throw up	2 (6.7%)	2 (6.9%)	2 (7.1%)
Feeling more or less hungry than you usually do	5 (16.7%)	1 (3.4%)	1 (3.6%)
Changes in taste	1 (3.3%)	0 (0.0%)	0 (0.0%)
Constipation (hard to poop)	0 (0.0%)	1 (3.4%)	0 (0.0%)
Diarrhea (watery, runny poop)	0 (0.0%)	0 (0.0%)	1 (3.6%)
**Patient-Reported Outcome Scores**			
Median PROMIS fatigue scale (IQR)	56.2 (50.7 to 59.9)	53.4 (45.1 to 58.5)	47.7 (41.7 to 54.0)
Median Total PedsQL 3.0 Acute Cancer Module Score (IQR)	70.8 (58.1 to 77.8)	74.1 (59.3 to 80.1)	71.8 (59.5 to 86.1)
Pain and hurt	81.3 (62.5 to 87.5)	75.0 (62.5 to 100.0)	87.5 (75.0 to 100.0)
Nausea	70.0 (56.3 to 80.0)	75.0 (60.0 to 85.0)	75.0 (55.0 to 90.0)
Procedural anxiety	66.7 (33.3 to 91.7)	66.7 (50.0 to 91.7)	70.8 (50.0 to 91.7)
Treatment anxiety	87.5 (66.7 to 100.0)	83.3 (66.7 to 100.0)	91.7 (64.6 to 100.0)
Worry	66.7 (50.0 to 83.3)	66.7 (50.0 to 83.3)	66.7 (56.3 to 91.7)
Cognitive Problems	65.0 (50.0 to 80.0)	70.0 (50.0 to 85.0)	70.0 (53.8 to 85.0)
Communication	75.0 (66.7 to 91.7)	66.7 (58.3 to 91.7)	79.2 (66.7 to 91.7)
Perceived physical appearance	75.0 (58.3 to 91.7)	91.7 (58.3 to 100.0)	91.7 (58.3 to 100.0)

Abbreviations: *IQR* interquartile range

* Score of 3 or 4 on a 5-point degree of bother Likert scale ranging from 0 to 4

Table 4 summarizes symptom documentation and intervention provision by time point. The most commonly documented symptoms at week 8 were “hurt or pain (other than headache)” (6, 20.7%), “feeling tired” (5, 17.2%) and “throwing up or feeling like you may throw up” (5, 17.2%). There was no documentation at either week 4 or week 8 for “changes in how your body or face look”, “mouth sores” and “changes in taste”. The most commonly treated symptoms were “throwing up or feeling like you may throw up”, “hurt or pain (other than headache)” and “headache”. The following symptoms were never treated at either week 4 or week 8: “changes in how your body or face look”, “feeling tired”, “feeling more or less hungry than you usually do”, “changes in taste” or “diarrhea”.

**Table 4:** Symptom Documentation and Intervention Provision by Time Point Independent of SSPedi Score

	Week 4	Week 8
	*n* = 29	*n* = 29
**Number Patients with Symptom Documentation (%)**		
Feeling disappointed or sad	1 (3.4%)	1 (3.4%)
Feeling scared or worried	2 (6.9%)	4 (13.8%)
Feeling cranky or angry	1 (3.4%)	1 (3.4%)
Problems with thinking or remembering things	0 (0.0%)	2 (6.9%)
Changes in how your body or face look	0 (0.0%)	0 (0.0%)
Feeling tired	6 (20.7%)	5 (17.2%)
Mouth sores	0 (0.0%)	0 (0.0%)
Headache	2 (6.9%)	1 (3.4%)
Hurt or pain (other than headache)	9 (31.0%)	6 (20.7%)
Tingly or numb hands or feet	2 (6.9%)	3 (10.3%)
Throwing up or feeling like you may throw up	7 (24.1%)	5 (17.2%)
Feeling more or less hungry than you usually do	3 (10.3%)	3 (10.3%)
Changes in taste	0 (0.0%)	0 (0.0%)
Constipation (hard to poop)	1 (3.4%)	0 (0.0%)
Diarrhea (watery, runny poop)	0 (0.0%)	3 (10.3%)
**Number Patients with Symptom Intervention (%)**		
Feeling disappointed or sad	2 (6.9%)	3 (10.3%)
Feeling scared or worried	2 (6.9%)	3 (10.3%)
Feeling cranky or angry	2 (6.9%)	3 (10.3%)
Problems with thinking or remembering things	1 (3.4%)	2 (6.9%)
Changes in how your body or face look	0 (0.0%)	0 (0.0%)
Feeling tired	0 (0.0%)	0 (0.0%)
Mouth sores	1 (3.4%)	2 (6.9%)
Headache	7 (24.1%)	4 (13.8%)
Hurt or pain (other than headache)	11 (37.9%)	7 (24.1%)
Tingly or numb hands or feet	3 (10.3%)	2 (6.9%)
Throwing up or feeling like you may throw up	17 (58.6%)	12 (41.4%)
Feeling more or less hungry than you usually do	0 (0.0%)	0 (0.0%)
Changes in taste	0 (0.0%)	0 (0.0%)
Constipation (hard to poop)	6 (20.7%)	3 (10.3%)
Diarrhea (watery, runny poop)	0 (0.0%)	0 (0.0%)

Additional file 1: Appendix 2 describes symptom documentation and intervention provision for symptoms stratified by the patient reporting that they were “not at all bothered” by the symptom (SSPedi score of 0), they were “a little” or “medium” bothered by the symptom (SSPedi score of 1 or 2) and they were “a lot” or “extremely bothered” by the symptom (SSPedi score of 3 or 4). In general, symptom documentation and intervention provision were not more common in those who reported more bothersome symptoms. Of note, among the 4 patients who reported they were severely bothered by feeling tired, symptom documentation was noted for one patient, and none received an intervention. Among the 4 patients who reported they were severely bothered by “throwing up or feeling like you may throw up”, symptom documentation was present for none and an intervention was provided for two.

Additional file 1: Appendix 3 shows the number of unplanned encounters per patient. Seventeen patients had at least one unplanned healthcare encounter during the eight-week study period, with these encounters being evenly divided across emergency department visits, unplanned clinic visits and unplanned hospital admissions.

Unsolicited qualitative feedback from patients and parents noted that SSPedi completion was easy and simple. Parents in cohort 2 noted that logging into SPARK on behalf of their child helped facilitate symptom reporting. One patient commented that completing SSPedi allowed her to reflect more on how she was feeling in terms of specific symptoms compared to when doctors asked her how she was feeling overall. One parent noted that participation in the study gave them a better understanding of how their child was feeling.

## Discussion

We found that after implementing interventions to enhance adherence with symptom reporting, three times weekly administration of SSPedi for eight weeks was feasible for pediatric cancer patients who were 8–18 years of age. It was also feasible to collect patient-reported outcomes at weeks 4 and 8. The main approaches identified to improve symptom screening were enabling pediatric patients to self-report symptoms by engaging with parents, providing approaches to remember the SPARK username and password, highlighting the potential benefits of clinicians receiving symptom reports and teaching patients to log-in to SPARK using their own device.

Our study is important because few pediatric cancer trials have evaluated longitudinal symptom reporting [20]. An important example is the PediQUEST study that included children with advanced cancer. In that study, patient-reported outcomes were completed weekly for those in clinic or on the ward, and by phone monthly for those not attending clinic [21]. Parents provided proxy-response if the pediatric patient refused to self-report. Another important study administered the PROMIS instruments longitudinally at three time points over one course of chemotherapy for pediatric cancer patients [22]. Assessments were obtained either in person or by telephone. A key distinction is that our approach uses an electronic platform to provide reminders to complete symptom reporting and thus, more closely mirrors clinical implementation. This transition from obtaining symptom reports using clinical research associates vs. using electronic platforms and more automated approaches will be a key consideration as we transition from research to practice.

We chose a three times weekly symptom screening frequency based upon the preferences of pediatric oncology clinicians participating in a cluster randomized trial of symptom screening [23]. The ideal frequency of routine symptom screening is not known. It is interesting that in Canada, among adult cancer programs, symptom screening typically either occurs infrequently or only with clinic visits [24]. Consequently, the concept of asking pediatric cancer patients to report symptoms three times weekly regardless of setting (home, clinic or inpatient) using an automated platform is novel. There are advantages to measuring symptoms at home as this is likely a better assessment of ongoing symptoms that require intervention.

Despite providing symptom reports to clinicians, the rates of symptom documentation and intervention provision were relatively low in this study. However, without a control group, the impact of symptom feedback to clinicians is not known. In our study, SPARK reports sent to clinicians included links to clinical practice guidelines to address the reported symptoms. It is possible that access to guidelines alone will not be sufficient to achieve practice change. We have hypothesized that adaptation of care pathways based on clinical practice guidelines may be an effective way to improve clinical practice guideline-concordant care [23, 25]. While describing symptoms was not a primary objective of this study, we also found that most patients had at least one severely bothersome symptom. This finding is concordant with other research, which found that symptoms including pain, fatigue, nausea and vomiting are common in pediatric oncology patients [22, 26–29].

The strength of our study was the utilization of standardized processes and procedures to measure chart review endpoints and the use of two reviewers to abstract symptom documentation and intervention provision. However, our study is limited by its conduct at a single center and its single group design. Feasibility of a single group trial does not guarantee feasibility of a randomized trial since patients and families may refuse randomization. One approach to overcome this issue could be a cluster randomized trial so that all patients at a given site would either be in the intervention or the control group.

In conclusion, three times weekly symptom reporting by pediatric patients with cancer for eight weeks was feasible. Mechanisms to enhance three times weekly symptom reporting were identified and implemented. Future studies of longitudinal symptom screening can now be planned.

## Supplementary Information

**Additional file 1: Supplementary file: Appendices. Appendix 1:** Feasibility Metrics by Cohort. **Appendix 2:** Symptom Documentation and Intervention Overall and by SSPedi Scores. **Appendix 3:** Number of Unplanned Healthcare Encounters (*N*=29). **Appendix 4:** Symptoms Associated with Unplanned Healthcare Encounters Documentation and Intervention Overall and by SSPedi Scores.

## Data Availability

The datasets used or analyzed during the current study are available from the corresponding author on reasonable request.
