# Supplementary material for: Feasibility of three times weekly symptom screening in pediatric cancer patients
Source: BMC Cancer. 2023 Jan 3;23:4. doi: 10.1186/s12885-022-10400-1 (PMC9809057; doi:10.1186/s12885-022-10400-1)
Supplement: Supplementary file 1 — Additional file 1: Supplementary file: Appendices. Appendix 1: Feasibility Metrics by Cohort. Appendix 2: Symptom Documentation and Intervention Overall and by SSPedi Scores. Appendix 3: Number of Unplanned Healthcare Encounters (N=29). Appendix 4: Symptoms Associated with Unplanned Healthcare Encounters Documentation and Intervention Overall and by SSPedi Scores. [file 12885_2022_10400_MOESM1_ESM.docx]

**Supplementary file: Appendices**

**Appendix 1: Feasibility Metrics by Cohort**

| **Completion and Feasibility Metrics** | **Cohort 1** | **Cohort 2** |
| --- | --- | --- |
|  |  |  |
| **Patients** | n=20 | n=10 |
| Median number of SSPedi completed per patient (IQR) | 21 (10 to 23) | 22 (17 to 25) |
| Number patients met feasibility threshold (≥ 60%) SSPedi completed | 11 (55%) | 9 (90%) |
| Median number SSPedi with at least one severely bothersome symptom* triggering email to HCPs (IQR) | 3 (1 to 8) | 3 (0 to 4) |
| Number patients with at least one severely bothersome symptom* at any time | 17 (85%) | 7 (70%) |
|  |  |  |
| **Reports** | n=319 | n=212 |
| Number of SSPedi reports with at least one severely bothersome symptom | 93 (29.2%) | 31 (14.6%) |

Abbreviations: IQR – interquartile range; HCP – healthcare professional

* Score of 3 or 4 on a 5-point degree of bother Likert scale that ranges from 0 to 4

**Appendix 2: Symptom Documentation and Intervention Overall and by SSPedi Scores**

|  | **Overall** | **SSPedi Score 0**  **(“not at all”)** | **SSPedi Score 1 or 2 (“a little” or   “medium”)** | **SSPedi Score 3 or 4 (“a lot” or “extremely”)** |
| --- | --- | --- | --- | --- |
|  | n=57 | n/N (%) | n/N (%) | n/N (%) |
| **Number Week 4 and Week 8 Assessments with Symptom Documentation (%)** |  |  |  |  |
| Feeling disappointed or sad | 2 (3.5%) | 1/43 (2.3%) | 1/13 (7.7%) | 0/1 (0.0%) |
| Feeling scared or worried | 6 (10.5%) | 2/44 (4.5%) | 4/12 (33.3%) | 0/1 (0.0%) |
| Feeling cranky or angry | 2 (3.5%) | 0/39 (0.0%) | 1/17 (5.9%) | 1/1 (100.0%) |
| Problems with thinking or remembering things | 2 (3.5%) | 1/39 (2.6%) | 1/18 (5.6%) | 0/0 (0.0%) |
| Changes in how your body or face look | 0 (0.0%) | 0/32 (0.0%) | 0/24 (0.0%) | 0/1 (0.0%) |
| Feeling tired | 11 (19.3%) | 4/15 (26.7%) | 6/38 (15.8%) | 1/4 (25.0%) |
| Mouth sores | 0 (0.0%) | 0/50 (0.0%) | 0/6 (0.0%) | 0/1 (0.0%) |
| Headache | 3 (5.3%) | 2/43 (4.7%) | 0/12 (0.0%) | 1/2 (50.0%) |
| Hurt or pain (other than headache) | 15 (26.3%) | 7/39 (17.9%) | 8/18 (44.4%) | 0/0 (0.0%) |
| Tingly or numb hands or feet | 5 (8.8%) | 0/39 (0.0%) | 4/16 (25.0%) | 1/2 (50.0%) |
| Throwing up or feeling like you may throw up | 12 (21.1%) | 4/35 (11.4%) | 8/18 (44.4%) | 0/4 (0.0%) |
| Feeling more or less hungry than you usually do | 6 (10.5%) | 3/24 (12.5%) | 3/31 (9.7%) | 0/2 (0.0%) |
| Changes in taste | 0 (0.0%) | 0/39 (0.0%) | 0/18 (0.0%) | 0/0 (0.0%) |
| Constipation (hard to poop) | 1 (1.8%) | 0/43 (0.0%) | 1/13 (7.7%) | 0/1 (0.0%) |
| Diarrhea (watery, runny poop) | 3 (5.3%) | 1/51 (2.0%) | 1/5 (20.0%) | 1/1 (100.0%) |
|  |  |  |  |  |
| **Number Patients with Symptom Intervention (%)** | n=57 | n/N (%) | n/N (%) | n/N (%) |
| Feeling disappointed or sad | 5 (8.8%) | 3/43 (7.0%) | 2/13 (15.4%) | 0/1 (0.0%) |
| Feeling scared or worried | 5 (8.8%) | 1/44 (2.3%) | 4/12 (33.3%) | 0/1 (0.0%) |
| Feeling cranky or angry | 5 (8.8%) | 3/39 (7.7%) | 2/17 (11.8%) | 0/1 (0.0%) |
| Problems with thinking or remembering things | 3 (5.3%) | 2/39 (5.1%) | 1/18 (5.6%) | 0/0 (0.0%) |
| Changes in how your body or face look | 0 (0.0%) | 0/32 (0.0%) | 0/24 (0.0%) | 0/1 (0.0%) |
| Feeling tired | 0 (0.0%) | 0/15 (0.0%) | 0/38 (0.0%) | 0/4 (0.0%) |
| Mouth sores | 3 (5.3%) | 2/50 (4.0%) | 1/6 (16.7%) | 0/1 (0.0%) |
| Headache | 11 (19.3%) | 7/43 (16.3%) | 3/12 (25.0%) | 1/2 (50.0%) |
| Hurt or pain (other than headache) | 17 (29.8%) | 8/39 (20.5%) | 9/18 (50.0%) | 0/0 (0.0%) |
| Tingly or numb hands or feet | 4 (7.0%) | 0/39 (0.0%) | 2/16 (12.5%) | 2/2 (100.0%) |
| Throwing up or feeling like you may throw up | 29 (50.9%) | 15/35 (42.9%) | 12/18 (66.7%) | 2/4 (50.0%) |
| Feeling more or less hungry than you usually do | 0 (0.0%) | 0/24 (0.0%) | 0/31 (0.0%) | 0/2 (0.0%) |
| Changes in taste | 0 (0.0%) | 0/39 (0.0%) | 0/18 (0.0%) | 0/0 (0.0%) |
| Constipation (hard to poop) | 9 (15.8%) | 8/43 (18.6%) | 1/13 (7.7%) | 0/1 (0.0%) |
| Diarrhea (watery, runny poop) | 0 (0.0%) | 0/51 (0.0%) | 0/5 (0.0%) | 0/1 (0.0%) |

**Appendix 3: Number of Unplanned Healthcare Encounters (N=29)**

| **Unplanned Encounter Type** | **n (%)** |
| --- | --- |
| Number total unplanned encounters per patient (%) |  |
| 0 | 12 (41.4%) |
| 1-2 | 9 (31.0%) |
| 3-5 | 4 (13.8%) |
| 6 or more | 4 (13.8%) |
| Number emergency department visits per patient (%) |  |
| 0 | 21 (72.4%) |
| 1-2 | 7 (24.1%) |
| 3-5 | 1 (3.4%) |
| 6 or more | 0 (0.0%) |
| Number unplanned clinic visits per patient (%) |  |
| 0 | 19 (65.5%) |
| 1-2 | 7 (24.1%) |
| 3-5 | 1 (3.4%) |
| 6 or more | 2 (6.9%) |
| Number unplanned hospital admissions per patient (%) |  |
| 0 | 20 (69.0%) |
| 1-2 | 6 (20.7%) |
| 3-5 | 3 (10.3%) |
| 6 or more | 0 (0.0%) |

**Appendix 4: Symptoms Associated with Unplanned Healthcare Encounters Documentation and Intervention Overall and by SSPedi Scores**

|  | Documentation with Any Unplanned Healthcare Encounter  (n=38) | Documentation During Emergency Department Visit  (n=10) | Documentation During Unplanned Clinic Visit  (n=14) | Documentation at Presentation of Unplanned Hospital Admission  (n=14) |
| --- | --- | --- | --- | --- |
| Feeling disappointed or sad | 0 (0.0%) | 0 (0.0%) | 0 (0.0%) | 0 (0.0%) |
| Feeling scared or worried | 0 (0.0%) | 0 (0.0%) | 0 (0.0%) | 0 (0.0%) |
| Feeling cranky or angry | 0 (0.0%) | 0 (0.0%) | 0 (0.0%) | 0 (0.0%) |
| Problems with thinking or remembering things | 0 (0.0%) | 0 (0.0%) | 0 (0.0%) | 0 (0.0%) |
| Changes in how your body or face look | 2 (5.3%) | 0 (0.0%) | 2 (14.3%) | 0 (0.0%) |
| Feeling tired | 8 (21.1%) | 4 (40.0%) | 4 (28.6%) | 0 (0.0%) |
| Mouth sores | 1 (2.6%) | 0 | 1 (7.1%) | 0 (0.0%) |
| Headache | 5 (13.2%) | 1 (10.0%) | 2 (14.3%) | 2 (14.3) |
| Hurt or pain (other than headache) | 11 (28.9%) | 3 (30.0%) | 4 (28.6%) | 4 (28.6) |
| Tingly or numb hands or feet | 0 (0.0%) | 0 (0.0%) | 0 (0.0%) | 0 (0.0%) |
| Throwing up or feeling like you may throw up | 8 (21.1%) | 3 (30.0%) | 1 (7.1%) | 4 (28.6) |
| Feeling more or less hungry than you usually do | 2 (5.3%) | 1 (10%) | 0 (0.0%) | 1 (7.1%) |
| Changes in taste | 0 (0.0%) | 0 (0%) | 0 (0.0%) | 0 (0.0%) |
| Constipation (hard to poop) | 1 (2.6%) | 0 (0%) | 1 (7.1%) | 0 (0.0%) |
| Diarrhea (watery, runny poop) | 4 (10.5%) | 1 (10%) | 1 (7.1%) | 2 (14.3%) |
